# Integration of molecular functions at the ecosystemic level: breakthroughs and future goals of environmental genomics and post-genomics

**DOI:** 10.1111/j.1461-0248.2010.01464.x

**Published:** 2010-06

**Authors:** Philippe Vandenkoornhuyse, Alexis Dufresne, Achim Quaiser, Gwenola Gouesbet, Françoise Binet, André-Jean Francez, Stéphane Mahé, Myriam Bormans, Yvan Lagadeuc, Ivan Couée

**Affiliations:** UMR 6553 ECOBIO, Centre National de la Recherche Scientifique, Université de Rennes 1, Campus de Beaulieubâtiment 14A, F-35042 Rennes Cedex, France

**Keywords:** Biodiversity, ecosystem functioning, environmental bioinformatics, environmental genomics, functional ecology, metagenomics, molecular ecology, systems biology

## Abstract

Environmental genomics and genome-wide expression approaches deal with large-scale sequence-based information obtained from environmental samples, at organismal, population or community levels. To date, environmental genomics, transcriptomics and proteomics are arguably the most powerful approaches to discover completely novel ecological functions and to link organismal capabilities, organism–environment interactions, functional diversity, ecosystem processes, evolution and Earth history. Thus, environmental genomics is not merely a toolbox of new technologies but also a source of novel ecological concepts and hypotheses. By removing previous dichotomies between ecophysiology, population ecology, community ecology and ecosystem functioning, environmental genomics enables the integration of sequence-based information into higher ecological and evolutionary levels. However, environmental genomics, along with transcriptomics and proteomics, must involve pluridisciplinary research, such as new developments in bioinformatics, in order to integrate high-throughput molecular biology techniques into ecology. In this review, the validity of environmental genomics and post-genomics for studying ecosystem functioning is discussed in terms of major advances and expectations, as well as in terms of potential hurdles and limitations. Novel avenues for improving the use of these approaches to test theory-driven ecological hypotheses are also explored.

## Introduction

All individuals and populations of individuals forming species live and forage within space and time limits. Understanding the interactions and functions of these organisms within their environment is the purpose of ecology, for which a large range of research strategies has been developed. However, exhaustive analysis of all the functional compartments in a given ecosystem presents a major challenge. Microorganisms (i.e. viruses, bacteria, Archaea and micro-eukaryotes), which are essential entities of biogeochemical cycles on the planetary scale (e.g. Falkowski *et al.* 2008), and represent approximately half of the total carbon contained in living organisms (Shively *et al.* 2001), are still considered as a black box in many ecological studies. Although we know more and more about the importance of microorganisms in nature, the current absence of crucial pieces of information is due not only to the tremendous diversity of genes, metabolisms and species of microorganisms but also to our incapacity to culture over 90% of them (Amann *et al.* 1995; Pace 1997). One of the major challenges facing ecology is therefore to obtain a holistic perception of ecosystems including a comprehensive understanding of microbial communities. Environmental genomics is one of the most promising approaches that can meet this challenge.

In the wider sense, environmental genomics in association with post-genomics (i.e. *transcriptomics* and *proteomics*; see the glossary for italicized terms) consists in studying large-scale sequence-based information obtained from a variety of environmental samples, at organism, population or community levels, in order to gain novel insights into evolutionary ecology, organism–environment interactions and processes of ecosystem functioning. As such information contains both synchronic (related to current functioning at a given point in time) and diachronic (related to historical and evolutionary dynamics) aspects, the deciphering of genomes, transcriptomes and proteomes is the most powerful and most large-scale approach to date that may link ecology, evolution and Earth history.

Environmental genomics and post-genomics are not restricted to bacteria and archaea community genomics, and can encompass studies of various other biological systems. For example: (1) mixed prokaryotic-eukaryotic microorganism communities, (2) small-size eukaryotes, especially pico- and nano-eukaryotes, (3) intricate multi-species networks of higher eukaryotic organisms, such as root mats or mixed-species insect swarms, (4) higher eukaryotic organism tissues containing their naturally associated parasitic or mutualistic symbionts and (5) non-model species that cannot be grown or raised under laboratory conditions. In other fields of research such as toxicology and ecotoxicology, environmental genomics generally refers to gene–environment or genome–environment interactions, thus including the study of model species, such as yeast or *Arabidopsis thaliana*, under strong environmental constraints (Teixeira *et al.* 2007) or from an evolutionary perspective (Delneri *et al.* 2008), or even studies of the human genome (Ballatori *et al.* 2003).

**Figure 1:**
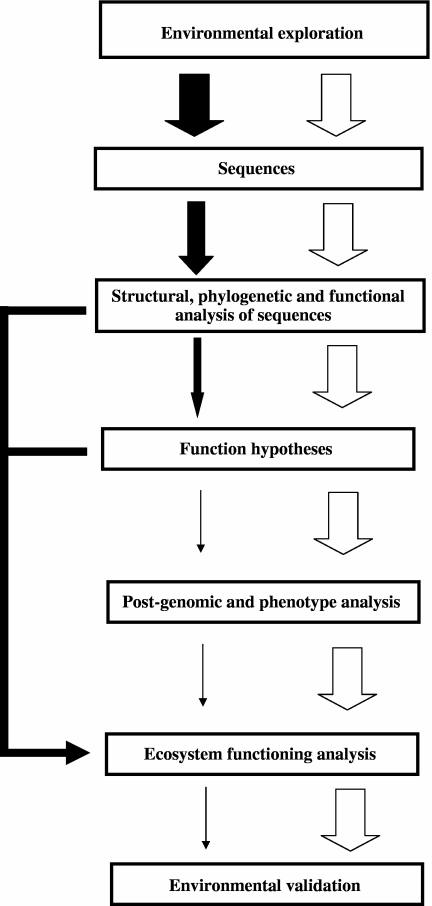
Real-life and ideal fluxes of analysis and information in environmental genomics. Current throughputs of analysis and information-processing are given as black arrows, whereas the ideal throughputs to be achieved are shown as white arrows. Arrow thickness reflects the efficiency of the analyses.

This review is focussed on environmental genomics and post-genomics in an ecological context, where analyses of large-scale sequence information can reveal how functions and signals are propagated and integrated at the different ecological levels – individual, population, community, ecosystem – and across various temporal and spatial scales. The aim of environmental genomics, transcriptomics and proteomics in an ecological context is to understand the ecosystem ‘dark matter’ (Marcy *et al.* 2007) after translation into nucleic acid and protein sequences (Fig. 1; Box S1), by taking advantage of the fact that these sequences convey functional information, interact with ecosystem parameters through environmental signalling and acclimation processes, and have been shaped by evolutionary pressures, thus offering a glimpse of past environments.

Given the great expectations associated with this recent field of research, we also discuss the validity of environmental genomics and post-genomics for studying ecosystem functioning, in terms of major advances and limitations, and then explore new avenues for improving these approaches to test theory-driven ecological hypotheses.

## Environmental genomics and the unification of different fields of ecology and biology

Clear connections exist between the hierarchic levels of ecological organization from individual to population to community to ecosystem. However, ecosystem ecology, which requires a mechanistic approach, is mainly based on physiological ecology (e.g. measurements of C, N or P fluxes). Ecosystem ecology is thus disconnected from the other ecological levels, and from the rest of ecology, although ignoring the question ‘who’s doing what?’ could be justified by the scale of the analysis. Along with this fact, and as pointed out by Fitter (2005), this dichotomy in ecology […] *has been framed in terms of functional redundancy* […], thus placing the ecological function as a cornerstone, while individuals are only considered as vectors of this ecological function. Hence, the consequences at the ecosystem level of changes at the population level are poorly known (Fitter 2005). Environmental genomics allows the diversity of organisms to be linked to the functions they display by providing the theoretical possibility of accessing at least partially every single species of a given ecosystem. As underlined by Ungerer *et al.* (2008), genomic approaches […] *offer new insights into higher-level biological phenomena that previously occupied the realm of ecological investigation only* […]. By removing previous dichotomies between ecophysiology, population ecology, community ecology, and phylogenetics on the one hand and ecosystem functioning on the contrary, environmental genomics along with genome-wide expression approaches greatly contributes to the merging of scientific fields and is a source of novel ecological concepts and hypotheses (see major breakthrough & new frontiers sections). However, linking diversity with the entire set of functions carried out by organisms in their natural habitat remains a major challenge.

## Integration of diversity and functions from molecular data

**Figure 2:**
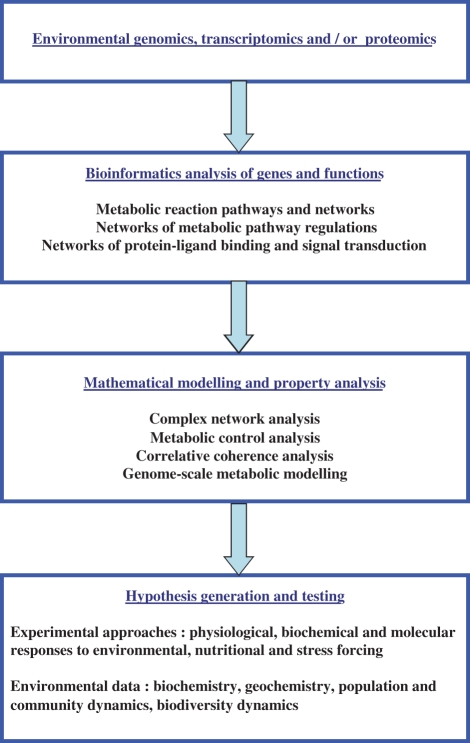
Mathematical modelling in environmental genomics analysis. Reconstructed networks from environmental genomics data (Box S2) can be analysed by various methods of mathematical modelling (Getz 2003; Feist *et al.* 2008; Westerhoff & Palsson 2008; Fuhrman 2009), that can assess and quantify their dynamic properties and generate hypotheses on community and ecosystem functioning. Hypothesis testing can then be carried out by experimental and environmental verification approaches, with the subsequent possibility of iterations between the different steps of the process. The main steps in this flowchart are derived from the description of the systems biology paradigm by Palsson (2006).

For over two decades, culture-independent molecular analyses have been used to analyse microbial community and population diversity, and also to study particular functions, such as denitrification or nitrogen fixation. In current environmental genomics studies, the metabolic and physiological potentialities of uncultured (micro)organisms are revealed by analyses of metagenomes (see Box S1 for details), i.e. the collection of genomes recovered from the same environmental sample, or from single-cell environmental genomes (see ‘major breakthroughs’ section). Despite analytical and technological limits (Table 1), advances in bioinformatics have improved the assembly of large fragments of genomes, the identification of RNA and protein-coding genes within these fragments and the determination of their biochemical and biological potential functions in complex mixtures of sequences from co-occurring organisms. The general aim of these analyses is to decipher taxonomic composition, metabolism, physiology and interactions in natural consortia of organisms in order to unravel evolutionary and ecological processes together with biotic interactions, as well as their changes over time and space. In other words, environmental genomics tackles the questions ‘who’s doing what, how, when and where?’ Furthermore, the correlations between the genetic and functional diversity of communities and environmental conditions can be used to integrate this sequence information into ecosystem processes (Box S1). However, it must be stressed that these approaches, although fruitful, ‘only’ provide hypotheses which must then be tested by other means (Figs. 1, 2). Analyses of genome sequences do not in fact reveal which functions are really expressed or identify the active organisms in a given process. The relevance of functional predictions and the validity of functional models based on genomics data can be improved by coupling environmental genomics with *(meta)transcriptomics* and *(meta)proteomics* approaches. It has also been shown that environmental genomics approaches can be coupled with direct probing or labelling of ecological processes. In an elegant work, Mou *et al.* (2008) used an experimental metagenomic approach to investigate the assimilation and mineralization of dissolved organic carbon by adding thymidine analogue bromodeoxyuridine as substrate in order to detect and extract the DNA of the individuals involved in the ecological process under study. The authors were able to elucidate the factors controlling heterotrophic communities (i.e. trophic interactions and physical conditions) and the rules controlling the assemblages of microorganisms within the studied ecosystem. This work presented convincing results arguing in favour of the ecological theory which predicts that heterogeneous environments are conducive to the establishment of generalist species with broad ecological niches (Kassen 2002). Other experimental metagenomic analyses using stable-isotope probing (Dumont & Murrell 2005) have greatly advanced our understanding of the actors in methane cycling (Cébron *et al.* 2007). Use of RNA stable-isotope probing has also led to new findings and hypotheses related to plant–microbe interactions and has highlighted that plants interact within their roots with many more microorganisms than previously believed (Vandenkoornhuyse *et al.* 2007). The selected studies above demonstrate that these approaches are not a mere technological tour de force. They provide novel insights into community structures and generate numerous functional hypotheses. The following section describes other striking examples of the application of environmental genomics to develop our understanding of ecosystem functioning.

**Table 1:** Advantages and limitations in environmental genomics and post-genomics

Stage of analysis	Advantages	Limitations
Sampling	No culture- or growth-related bias	Spatio-temporal heterogeneity
	Direct environmental sampling; large multi-species sampling; large multi-tissue sampling	Cost of representative or exhaustive sampling
	Analysis of complex experimental designs involving populations and communities	Careful ecological assessment of environmental sampling and of experimental designs
	Possible long-term storage of DNA, RNA, or protein samples	Availability of reliable protocols for the extractions of nucleic acids and proteins
Sequencing	High-throughput technologies for DNA, RNA and proteins	Possibilities of sequencing bias; poor sequencing of less-represented genomes
	Decreasing cost of sequencing and mass spectrometry	Cost of sequencing for large sample collections, in relation to the exhaustiveness of sampling
	Long-term public databases	Exponential increase of the amount of sequence data; cost and maintenance of database infrastructure
Information processing and functional analysis of organisms, communities and ecosystems	Biodiversity and phylogenetic analysis	Taxonomic bias in databases
	Functional profiling of naturally occurring organisms and communities	Assembly of short genomic fragments giving a partial view of organismal functional capacities
	Link function and diversity and answer the question ‘who is doing what?	Functional bias in database; computational demand for bioinformatics analyses; poor quality of annotations and amplification of annotation errors
	Discovery of novel ecologically relevant functions	Functional inferences from genomics data in the absence of transcriptomic and/or proteomic data; biased conclusions on the basis of apparent absence of function
	Identifying links between diversity, functional changes and environmental variables	Experimental bottleneck of functional characterization of new genes
	Evolvability of genomics data analysis through improvement of annotations	Computational cost of re-annotating sequences
	Re-analysis of genomics data in the light of novel environmental data	Comprehensive environment variable surveys;environment variable databases;environment-dedicated bioinformatics tools;exponential increase of environmental data; increased complexity of the comparison between environmental data and genomics data
	Comparison of present-day ecosystem functioning with earth history and paleo-ecosystem functioning Combination of synchronic and diachronic analysis	
	Identifying links between diversity, functionalchanges and environmental variables	Confusing the reality of ecosystem functioning with the reconstructed image from environmental genomics

## Major breakthroughs of environmental genomics

One of the most innovating aspects of environmental genomics is the capacity to predict new functions and to infer relationships between functions, whether novel or not, and particular species or specific communities. A classic example is the discovery of a new class of light-driven proton pumps in uncultured marine proteobacteria (Béjà*et al.* 2000). These proteins, named proteorhodopsins, might sustain a photoheterotrophic lifestyle in many planktonic bacteria and archaea species (de la Torre *et al.* 2003; Frigaard *et al.* 2006) inhabiting various sunlit aquatic environments (Béjà*et al.* 2001; Sabehi *et al.* 2003; Venter *et al.* 2004; Atamna-Ismaeel *et al.* 2008). However, the physiological and ecological roles of every type of proteorhodopsin need to be fully described (Fuhrman *et al.* 2008).

The strength of environmental genomics was also shown when mesophilic Crenarchaeota could be linked to ammonium oxidation. Few specific bacterial groups were known to use ammonium as an energy source. Parallel application of environmental genomics approaches to marine plankton and soil samples led to identification of genes encoding for an ammonium monooxygenase on genomic fragments affiliated to Archaea (Venter *et al.* 2004; Treusch *et al.* 2005). In an impressive follow-up study, Leininger *et al.* (2006) not only showed that one subgroup of mesophilic Crenarchaea actively catalyses ammonium nitrification but also established that archaeal *amoA* genes were much more abundant than the corresponding bacterial genes in different soil samples, thus suggesting that they are major players in ammonia oxidation in diverse soil ecosystems. This discovery produced a downright jump-start for an enormous number of studies of Crenarchaeota in other terrestrial and marine environments, most of the results indicating the prevalence of Archaea over Bacteria in this first step of nitrification. The hypothesis that Archaea play an important role in the overall N-cycle was therefore considerably strengthened. These are two impressive examples of how the detection of key protein-coding genes on a genomic fragment can challenge long-lasting ecological paradigms.

In the above studies, the authors sequenced long fragments of DNA bearing taxonomically or functionally informative genes. In contrast, community-centered approaches, followed for instance by Tyson *et al.* (2004) and Venter *et al.* (2004), have demonstrated the possibility of inferring the structure and the potential activity of microbial assemblages using *shotgun sequencing*.

The biofilm analysed by Tyson and co-workers flourishes at the surface of highly acidic, metal-rich drainage waters in an iron mine. Because of the very reduced biodiversity in this extreme environment, the authors were able to reconstruct two near-complete genomes and they deduced the potential biological functions of the organisms in the biofilm in relation to water chemistry. In particular, they were able to hypothesize that bacteria of the *Leptospirillum* group III, which were relatively sparse in the biofilm, were probably the only group of N_2_-fixing organisms and therefore the single possible point of entry of nitrogen in the biofilm.

Environmental genomics tools have also been applied to ecosystems harbouring more diverse microbial communities. In one of the largest environmental genomics study ever undertaken, Rusch *et al.* (2007) produced a total of 7.7 million reads from samples of surface waters collected during the Global Ocean Sampling expedition off the eastern American coast, in the Gulf of Mexico, the Panama canal and in the eastern part of the equatorial Pacific Ocean. Despite a strong sequencing effort, 53% of the reads remained unassembled, which could be ascribed to the high levels of diversity within the samples. However, despite this high level of genetic polymorphism, this impressive dataset was dominated by very few genera of bacteria such as *Pelagibacter*, *Prochlorococcus and Synechococcus,* which were found at many sites along the transects. Two other abundant genera, *Burkholderia* and *Shewanella,* only appeared in the Sargasso sea (Venter *et al.* 2004). These five genera were also found to be among the most abundant in the dataset when 16S rRNA sequence clusters were used to characterise the diversity. A large fraction of the diversity fell within ribotypes, with the presence of distinct populations in different environments. Likewise, computations of the similarities between community genomes were used to assess genetic distances between sampled environments. Samples from unique habitats such as a hypersaline pond and a freshwater lake were the most distant in terms of genomic composition whilst similar habitats such as the Sargasso sea or tropical open ocean waters contained more similar microbial metagenomes.

Environmental constraints exert a strong selection pressure on living (micro)organisms. These factors drive the selection of guilds that are best adapted for habitat colonisation. Thus, application of environmental genomics on a ‘global’ scale (e.g. through sampling along a gradient of environmental fluctuation or through comparison of different ecosystems) offers an unprecedented way of linking environmental parameters with the specific and functional diversity of microbial assemblages (see also Tringe *et al.* 2005; Dinsdale *et al.* 2008).

Metagenomic studies have offered a broad view of the organization of genetic diversity in various microbial communities as well as insights into the metabolism of their dominant members. However, the paucity of fully assembled genomes from metagenome sequencing has hampered our ability to link diversity and functions. The need to target specific groups of organisms in an environmental sample has led to the development of numerous methods and protocols for isolating populations ranging from a few thousand cells to only one cell and for obtaining enough DNA template for sequencing (Rodrigue *et al.* 2009; Woyke *et al.* 2009). Recently, Zehr *et al.* (2008), by deciphering the genome sequence of a new group of unicellular nitrogen-fixing marine cyanobacteria dubbed UCYN-A, have provided an excellent example of how the combination of isolation techniques and environmental genomics helps to link ecosystem functioning with the genetic makeup and metabolic features of organisms. UCYN-A cyanobacteria were first detected through the amplification of transcripts of the *nifH* gene (dinitrogenase reductase subunit of nitrogenase; Zehr *et al.* 2001) in environmental samples. Unlike other unicellular diazotrophic cyanobacteria, UCYN-A cyanobacteria express the *nifH* gene during daytime when oxygen production by photosystem II (PSII) inhibits nitrogen fixation (Church *et al.* 2005). Despite repeated efforts, no member of this group could be maintained in culture. The authors used flow cytometry to isolate about 5000 cells from a natural population of the UCYN-A group and subjected the genomic DNA to isothermal whole genome amplification and pyrosequencing. As expected for a diazotroph, the UCYN-A metagenome encodes a complete nitrogen fixation pathway. Surprisingly, although numerous sequences of Photosystem I genes were detected, no genes coding for the PSII proteins were found. The authors provided strong evidence that cyanobacteria of the UCYN-A group do not possess a complete photosynthetic apparatus and also seems to lack all the genes necessary for CO_2_ fixation. Thus, the UCYN-A group appears to be the sole known cyanobacterial lineage unable to produce oxygen. This would explain how UCYN-A cyanobacteria concomitantly perform N_2_ fixation and photosynthesis. Several studies had suggested that members of the UCYN-A group were abundant in oceans and might contribute markedly to biological nitrogen fixation (Montoya *et al.* 2004). The inability of some marine diazotrophs to fix CO_2_ will certainly require a refinement of established models of N and C cycling in oceans as it deviates from the stoichiometrical relationships previously assumed for biological N fixation and photosynthetic C incorporation (Mahaffey *et al.* 2005).

Environmental genomics has become a standard approach in the study of aquatic habitats, owing to their relative simplicity. In comparison, soils and sediments appear to be more spatially heterogeneous and phylogenetically diverse. Estimates of soil diversity are often in the range of hundreds to thousands of microbial species per gram of soil (Torsvik *et al.* 2002). Soil and sediments are often considered to constitute one of the largest reservoirs of microbial diversity on Earth. Notwithstanding the difficulties of obtaining representative samples or limitations associated with DNA extraction and purification (Table 1), sequencing of metagenomes from soil communities also requires much greater effort to obtain significant sequence coverage. Consequently, terrestrial habitats have mainly been targeted by metagenomic studies in the prospect of finding new molecules of biomedical or agricultural interest (Daniel 2005). International programs such as TerraGenome have been started with the aim of sequencing the metagenomes of reference soils (see http://www.terragenome.org/).

The use of high-throughput sequencing technologies has also led to tremendous progress in understanding the intricate associations between symbiotic microorganisms and their eukaryotic hosts. Woyke *et al.* (2006) described the functioning of a complex symbiosis between the marine oligochaete *Olavius algarvensis* and a microbial consortium consisting of two sulphur-oxidizing gammaproteobacteria and two sulphate-reducing delta-proteobacteria. The worm is characterized by the complete absence of a digestive apparatus and a reduced excretory system. Thus, nutrition of the host, as well as the degradation of toxic by-products of its metabolism, is entirely dependent on the activity of the bacterial consortium. Analysis of the metagenomic data provided valuable insights into the metabolism of the different bacterial partners and into the network of interactions established between the worm and its symbionts. The host is supplied with C, N, S and P compounds by the symbiotic bacteria, and host organic osmolytes and waste products are used as C and N sources for symbiont metabolism. Analysis of the protein-coding genes of the symbionts has confirmed the existence of syntrophic cycling of sulphur elements between the sulphur-oxidizing and the sulphate-reducing symbionts.

Finally, organism-centered studies of isolable multicellular eukaryotes (Martin *et al.* 2008; Vera *et al.* 2008; Rasmussen & Noor 2009) have shown the usefulness of environmental genomics for analysing such organisms in their ecological and evolutionary context. Altogether, these examples of function-, organism-, community- or environment-centered approaches shed light on how environmental genomics and post-genomics allow the integration of molecular data with ecological metrics and open new windows on the complex interplays between genomes, phenotypes, populations and environment. All these results, which have already induced advances in ecology, are based on a battery of bioinformatics tools (see Box S2 for details) to analyse sequence data. However, there are still limitations, which are discussed below, along with recommendations to avoid mis-analyses and mis-interpretations.

## Current limitations of environmental genomics functional integration

### Sampling and sequencing

Technological and conceptual limitations of environmental genomics (Table 1) are not trivial, and require thorough consideration to further improve analyses. Confrontation with various environmental samples (such as seawater, freshwater, soils, sediments, bacterial mats, plant and animal tissues) has resulted in the considerable improvement of extraction protocols and methods, and of sample preparations, which must be environment-compatible, contamination-free, non-degradative, non-combinatorial, and complete. Considerable progress has also been made in the quality of massive sequencing in terms of throughput, cost, read length, and read quality. Current sequencing methods can generally yield deep and representative environmental sequences of high quality. Moreover, these methods are constantly improving and bioinformatics analysis of sequences is constantly reducing sequencing noise and bias (Quince *et al.* 2009). However, the quality and representativity of sequencing may remain hampered by the complexity of some environmental samples, in terms of organism diversity and abundance as well as size and composition (e.g. percentage of repeats) of the individual genomes.

### Gene identification and functional characterisation

The first task of finding genes in environmental genomics or metagenomics data is sometimes compounded by the great diversity of genomes that is revealed and by the myriad novel genes they contain (Table 1). Whereas gene identification has become less and less problematic for bacteria and archaea genomes, the difficulties must not be underestimated in the case of higher eukaryotic genomes (Levasseur *et al.* 2008) due to the modular nature of eukaryotic genes and to the short sequences produced by second-generation sequencing platforms which complicate the prediction of *open-reading frames*.

Another major challenge in environmental genomics is the subsequent step of correctly identifying functions on the basis of sequence data. Classically, the identification of gene functions is heavily dependent on comparisons, using standard tools such as BLAST (Basic Local Alignment Search Tool, Altschul *et al.* 1997), with sequences from other organisms or metagenomes present in genome databases such as GenBank. The inference of gene function is then derived from functional annotations of these similar sequences. Bioinformatics analyses are thus becoming a major bottleneck in environmental genomic studies (Fig. 1), as the production of sequences outpaces the computational capacities available in most laboratories. Moreover, as highlighted by Palsson (2006), “*it should be emphasised that every gene annotation based on in silico methods is hypothesised and such annotation is subject to revision, until the gene has been cloned, expressed, and the function of the gene product directly evaluated*”. Thus, most *bona fide* annotations are derived from genes of model organisms, where biochemical analysis and reverse genetics can readily be carried out. Furthermore, the sequenced organisms available in databases represent a small and strongly biased subset of the biodiversity revealed by cultivation-independent methods. However, it is worth noting that several recent initiatives such as the Moore Foundation Marine Microbial Genome Sequencing Project, the Genomic Encyclopedia of Bacteria and Archaea Project, or the Fungal Genome Initiative will contribute to improve the list of sequenced organisms and to obtain a better coverage of the known biodiversity.

This duality between the great phylogenetic diversity of environmental genes (Yooseph *et al.* 2007) and the limited number of well-characterised genes in the databases is likely to result in high proportions of genes with ‘unknown’ or ‘hypothetical’ functions in environmental genomes. This may also cause a strong bias towards identification of the best-known, and maybe most straight-forward, functions, such as those related to central metabolism. Finally, numerous causes of incorrect annotations in model species have been identified (Galperin & Koonin 1998). This is why some authors have voiced concern that comparison of environmental genomes with imprecise or erroneous annotations in databases may lead to exponentially amplified errors and inappropriate functional predictions (López-García & Moreira 2008).

### The concept of function and the difficulty of function assignment

Most studies of gene-function relationships have focussed on the cell and organismal levels. Even at these levels, the difficulty of precisely defining the multi-faceted concept of function has been emphasised (Danchin *et al.* 2004) and gene functions may be more complex than those hypothesised from database annotations. A well-annotated gene, with a well-defined function, may yield various products through alternative splicing and post-translational modifications, and/or multi-functional products. For instance, a gene may code for multiple enzymatic activities, with multiple subcellular localizations (Silva-Filho 2003), or with combined enzymatic and regulatory functions (Takeda *et al.* 2009). Complete understanding and annotation of gene product functions are therefore extremely difficult to achieve (Danchin *et al.* 2004).

Moreover, many annotations that are based purely on sequence homology are likely to be incorrect, since biochemical characterization of gene products previously identified by similarity searches has often yielded surprises, especially in terms of ligand/substrate specificities or of subcellular targeting. Conversely, an apparent absence of gene families on the basis of homology searches does not necessarily mean an absence of function since independent emergence of catalytic processes can occur in independent protein phylogenetic backgrounds, thereby creating sets of analogous enzymes (Galperin *et al.* 1998). Finally, whereas *homologousidentification* can be extremely precise on the basis of short sequences, as in the identification of short expressed tags vs. genome data from the same organism, *heterologous identification* of unknown genes vs. gene databases from more or less related organisms can be hazardous. Thus, as an exaggerated example, BLASTX analysis (search of protein databases for all the translated possibilities of a DNA sequence) of the complete gene sequence of *Nicotiana tabacum* ornithine decarboxylase (polyamine biosynthesis pathway) *versus* the *Arabidopsis thaliana* protein database yields a significant identification with diaminopimelate decarboxylase (lysine biosynthesis pathway). This instance of heterologous mis-identification between related species may be ascribed to the fact that *Arabidopsis thaliana* lacks an archetypal ornithine decarboxylase (Hummel *et al.* 2004).

It is clear that all the above-described situations are likely not only to occur but also to be compounded at the ecosystem level where multiple environmental variables drive the expression of gene functions and direct the role played by organisms in ecosystem processes. Furthermore, our ability to determine the links between biodiversity and ecosystem functioning might be hampered by the importance of horizontal transfers of protein-coding genes – for instance through viruses or plasmids – between phylogenetically distant Bacteria and Archaea (Koonin & Wolf 2008).

### Genome–environment interactions and the plasticity of gene expression

Although identification of a given function at the gene level may indicate selection of this gene in the organisms present in the ecosystem, it does not give information on the patterns of gene expression. In other words, there are always important differences between who is there in the ecosystem and who is at work in the ecosystem. As far as possible, genomics data must be complemented with transcriptomics or proteomics data, which correspond to measurements of steady-state levels of transcripts or proteins (Box S2; Fig. 2).

Although its adaptive value has been subjected to criticism (Feder & Walser 2005), mRNA expression is an important step in gene-to-functional protein expression (Stranger *et al.* 2007), and an important response to the perception of environmental clues (Hummel *et al.* 2004). Improvement of RNA isolation and application of massive sequencing to the analysis of cDNA from environmental samples (Frias-Lopez *et al.* 2008) or non-model species (Vera *et al.* 2008) have circumvented the limitations of *DNA array technologies*. In spite of some successful applications (Parro *et al.* 2007), DNA array technologies cannot be readily applied to most environmental samples, since they imply *a priori* knowledge of the species and communities under investigation. It must be kept in mind however that environmental transcriptomics suffers from some drawbacks, such as the variable half-lives of mRNA, and the fact that, in bacteria and archaea, mRNAs represent a small proportion of the total RNA and cannot be enriched by poly-dT affinity, since they lack the polyA tail found in eukaryotic mRNA. Moreover, functional characterization of cDNAs is confronted with the same limitations of annotation as those described above for gene function analysis (Table 1). Finally, transcriptomics generally gives a comprehensive view of expression levels across the individuals of the sampled population (Stranger *et al.* 2007). More detailed analysis of environmental transcriptomics data should eventually take into account the impact of individual genetic variations on gene expression (Stranger *et al.* 2007).

Analysis at the protein level may provide the most representative snapshots of organism or community functionalities. Proteomics and metaproteomics approaches have indeed been carried out with success on environmental samples (Ram *et al.* 2005). Nonetheless, reliable extraction of proteins from natural environments can be more challenging than for nucleic acids, especially in terms of the quality and quantity of the sampled proteomes. High throughput analysis of metaproteomes can be carried out by mass spectrometry, which however requires comparison with databases containing gene sequences originating from the same organisms or from very closely related organisms, as mass spectrometry data are very sensitive to changes in protein sequences. Thus, metaproteomics studies must be coupled to metagenome sequencing to detect significant numbers of protein matches (Ram *et al.* 2005).

### From environmental genomics to environmental phenotypes

As most metabolic and functional schemes of ecosystem functioning are dependent on heterologous comparisons with databases containing significant numbers of *in silico* annotated genes, such schemes should be clearly labelled as hypothetical (Fig. 2). This hypothetical nature does not undermine the core value of such analyses, but should be taken as an incentive to validate hypotheses and integrate these hypothetical schemes into further ecosystem-level studies. In other words, caution must be taken not to indulge in direct integration of sequence analysis, which may short-circuit important validation steps (Fig. 1). Moreover, due to regulatory, biochemical and supramolecular interactions, the number and scope of organism and ecosystem functions derivable from a single genome or from community genomes does not scale with the mere catalogue of genes contained in those genomes.

The identification of new environmental genes should be followed by further functional, biochemical, and physiological characterization. This can first be carried out on candidate genes, selected on the basis of their outstanding interest or representativity in relation to ecosystem knowledge. This was the case for proteorhodopsin genes. They were identified in analyses of environmental DNA, and their products were biochemically characterised after *over-expression* (Béjà*et al.* 2000). Furthermore, environmental genomics data can be complemented with laboratory organism-centered approaches, not only in the case of isolable multicellular eukaryotic organisms, but also in the case of microbial communities. Thus, enrichment cultures and the cultivation of selected microbial strains may be useful for further genomic and physiological characterisation (Giovannoni *et al.* 2005) or to test important physiological and ecosystemic hypotheses (López-García & Moreira 2008). In this context, important progress has been made to develop culture protocols and media to cultivate recalcitrant microorganisms of ecological interest (Ben-Dov *et al.* 2009).

More generally, environmental genomics results must be critically confronted with ecological ecosystem knowledge (Mou *et al.* 2008; Zehr *et al.* 2008) and/or tested through modelling procedures (Röling *et al.* 2007). Procedures for environmental validation, corresponding to a kind of ecosystem phenotype characterization, should be better defined, in the same way that model species genomics should be complemented with organism phenotype characterisation (Fig. 2). However, it may be extremely difficult to carry out high-throughput post-genomics functional characterisation, such as protein over-expression and biochemical analysis, mutant-based gene/function analysis or natural variation-based gene/function analysis, in the context of environmental genomics (Wullschleger *et al.* 2007). However, it has to be stressed that bioinformatics approaches and tools can yield broad and useful information, especially functional information, even with a genome coverage as low as 0.1X (Rasmussen & Noor 2009), when long enough sequence tags are obtained from random pyrosequencing. This is true even for communities of organisms that do not correspond to any available genomic sequence in the databases. Moreover, novel ideas and methods are constantly improving the relevance of environmental genomic analyses to address ecological questions.

## Improvement of genomics approaches from an ecological point of view

### The importance of ecological and evolutionary criteria for functional identification

The difficulties of homology-based functional identification have been recognized for some time, but various improvements using protein domain detection and gene context approaches (Singh *et al.* 2009) have been made. Phylogenetic analyses have been particularly valuable in going beyond basic homology comparisons and accounting for the evolutionary history of genes (Levasseur *et al.* 2008). Thus, combinations of phylogenetic tree construction, integration of experimental data and differentiation of *orthologs* and *paralogs*, have been proposed to address annotation errors. As a result, a number of software platforms and databases have been developed recently (see Box S2). These enable phylogenetic analysis and utilisation of gene clusters, such as COGs (clusters of orthologous groups; Tatusov *et al.* 2003), to infer gene function by superimposing experimental information on the phylogenetic trees (Levasseur *et al.* 2008). The use of phylogenetic data for functional reconstruction from environmental genomics is particularly interesting in the light of relationships between community phylogenetic structure and ecosystem processes (Prinzing *et al.* 2008). However, the quality of this kind of phylogeny-based analysis is strongly dependent on the scope of the initial phylogenomics database and on relationships between the environmental species under study and the set of species present in the databases.

### The importance of bioinformatics and statistical controls

Given the unfinished status of gene and protein databases, it may be important to develop experimental bioinformatics controls, especially when the species in the environmental genomics data do not have phylogenetically related counterparts in the databases. Thus, controls can be carried out with artificially-reconstructed genomes (Yang & Bennetzen 2009) or communities (Quince *et al.* 2009). In robustness controls, a known genome of a control species could also be re-analysed by comparison with gene and protein databases from which this given species, its genus, or its family would be artificially removed. This approach could be used to estimate the accuracy of functional assignments when an unknown genome is compared with phylogenetically unrelated genomes, and thus to select the most robust functional assignments. Environmental genomics approaches often imply the parallel comparative analysis of various samples corresponding to gradients of ecological factors, such as light, salinity, or anthropic pressure (Raes *et al.* 2007; Dinsdale *et al.* 2008; ). The complexity of environmental genomics data therefore requires the specific development and/or adaptation of statistical analysis tools as described in Rodriguez-Brito *et al.* (2006).

### Expected improvements of functional annotations and genome assembly

As described above, a great number of functional annotations are hypothetical and subject to revision. Conversely, continuous revision can be expected to improve environmental genomics data analysis. However, systematic and standardized processes for database revision are still lacking, and need to be developed for all the different genomics approaches, whether model-species-based or environmental, in order to avoid possible erroneous revisions. Moreover, novel methods, such as those taking into account not only the nature of direct gene products but also regulatory interactions, protein-protein interactions, and protein-metabolite interactions (Palsson 2006), are likely to improve annotations. Developing comparisons of metagenomics data with metatranscriptomics and metaproteomics data can also be expected to improve *in silico* identification of genes and annotations. Finally, full and accurate annotation of model species genomes, corresponding to different major phyla, remains to be carried out and may further improve environmental genomics data analyses. However, the diversity and variability encountered in environmental genomics data may eventually surpass the range of model species genomics data and even modify the very concept of species and of model species (Medini *et al.* 2008). Moreover, model species databases will be progressively complemented with databases for single-species genomes of ecological interest, especially if single-cell genomics (Marcy *et al.* 2007; Rodrigue *et al.* 2009; Woyke *et al.* 2009) can be developed in an ecological context. These environmental genomics data on single species, obtained through direct sampling of individuals, cultivation or single-cell approaches, will be extremely useful not only for annotation but also to assemble metagenomics data.

### Further analysis of the complete wealth of environmental genomics data

In the same way that they can be re-analysed in the light of improved annotations, stored environmental genomics data can be re-analysed to extract meaningful new information. For instance, the comparative analysis of *promoter sequences*, which are involved in gene expression regulation, has been extremely limited in the case of environmental genomics data. Promoter sequences involve consensus sequences and *regulatory cis-acting elements* that can be highly conserved across species or highly variable, depending on evolutionary constraints and selection pressures (Zhu & Snyder 2002). Furthermore, databases of promoters are being developed (Zhu & Snyder 2002). Therefore, it could be possible to classify gene sets from environmental genomics data according to the cis-acting regulatory elements that are present in their promoters, thereby generating classes of co-activated or co-inhibited genes. Insofar as cross-species consensus sequences are available for use, such classification could point to co-regulated genes at the community level. Moreover, such information on co-regulation at the ecological level could lead to experimental verification using *ChIP-on-chip approaches* on the proteins that regulate these networks of co-regulated genes (Buck & Lieb 2004). Similarly, it will be possible in the future to carry out deeper analyses of environmental genomics data for other regulatory levels, such as the generation of multiple transcripts from a single gene (Méreau *et al.* 2009) or the systematic analysis of regulatory RNAs (Shi *et al.* 2009). Finally, in parallel to environmental genomics, the miniaturization and automation of sensors and probes have also resulted in the development of powerful analytical tools that make it possible to carry out high-frequency temporal, as well as proximal, monitoring of natural habitats. Such tools are essential to monitor environment variables at scales of time and space relevant to community activities and molecular functions. Analytical microsensors are able to monitor fine variations or gradients of various physico-chemical parameters (Krawczyk-Barsch *et al.* 2008). Likewise, isotopic (nanoSIMS) and microscopic techniques (FISH, TEM) can measure the activities of (micro)organisms in their habitats (Dekas *et al.* 2009). Progress has also been achieved in the setting-up of controlled experiments, in which the complexity of communities and the geochemical environments can be manipulated. The use of environmental genomics approaches that combine accurate monitoring and experimentally controlled environments may contribute to build appropriate models of ecosystem functioning (Fig. 2).

### Present and future importance of mathematical modelling for environmental validation

Environmental genomics data are complex in scale and scope. Even the pivotal task of inferring community-level functions from individual functions of genes requires the parallel analysis and integration of hundreds or thousands of genes and individual functions, and an understanding of their functional and regulatory interactions. For the reasons given above, genomics-based data must be compared and integrated with higher-level environmental data, such as experimental data or fluxes of biogeochemical cycles. The richness and complexity of these data raise the problem of transforming functions into equations. However, it is important to be able to describe reconstructed functional networks mathematically, in order to analyse their properties in greater detail (Palsson 2006). Mathematical properties can be used to generate functional hypotheses (Fig. 2) through complex networks analysis (e.g. Fuhrman 2009), metabolic control analysis (Westerhoff & Palsson 2004), correlative coherence analysis (Getz 2003), or genome-scale metabolic modelling (Feist *et al.* 2008). These hypotheses can then be tested experimentally or tested for their fit to environmental data, such as geochemical fluxes, biodiversity fluctuations, or biomass production. Finally, models of reconstructed networks can be improved by iterative interactions between modelling, experimental results and ecosystemic data (Fig. 2).

## New frontiers of environmental genomics

The present state-of-the-art shows that environmental genomics has already generated new concepts and tackled questions that were impossible to address before. Improvement of multidisciplinary integration of bioinformatics, genetics, statistics, physiology, ecology, and evolutionary sciences, is likely to raise further questions and to offer the possibility to reinvestigate existing paradigms.

Environmental genomics is leading to a better understanding of diversity at different ecological scales ranging from population to ecosystem by demonstrating that the environmental gene pool is several orders of magnitude greater than previously believed (Yooseph *et al.* 2007). It is clear, from these findings, that the availability of one complete genome sequence for each described taxon would be insufficient to explain the complexity of species (Medini *et al.* 2008). Despite the fact that species are considered as fundamental units of biology and are thus as important as the cell or individual, the definition of a species and the adoption of a unified species concept is still under debate, although interesting essays on this topic have been published (Mishler & Brandon 1987; de Queiroz 2007). Ribosomal RNA gene analyses have been long considered as sufficient tools to describe diversity because (1) these genes are shared by all living organisms, (2) they contain robust phylogenetic information and (3) they are useful, easy-to-apply tools for application of the phylogenetic species concept (Mishler & Brandon 1987). Environmental sequencing has recently provided a global ‘one-does-all’ method providing a deep insight into the molecular list of all the sampled (micro)organisms, and describing the genes and functions displayed in more or less complex communities. From this, it becomes possible to consider a genome as a trait and to delimit species as ‘*separately evolving metapopulation lineages (or, more properly, segments thereof)’* (de Queiroz 2007) by analysing this trait rather than core genes, such as ribosomal RNA genes. It also has to be stressed that the adoption of an explicit species concept directly affects the actual assessment of diversity and thus the fit of (1) models of community dynamics and (2) theories of species assembly. The use of the genome as a trait to describe a species could involve, among other criteria, *gene synteny* and the level of similarity. However, at present, this can be envisaged only for small-genome organisms, such as bacteria, archaea and some eukaryotes.

Besides these considerations, novel fields of research that cannot be studied by other means than environmental genomics are now open to investigation. Pioneer papers, at the intersection of ecology and evolutionary biology, have paved the way for the genomics of co-evolution including mutualism, symbioses and parasitism. For instance, Martin *et al.* (2008) analysed mycorrhizal symbiosis and provided important insights into the behaviour and capacities of the fungal symbiont. In a similar line of research, the behavioural evolution and capacities of insect heritable bacteria have been explored (e.g. Moran *et al.* 2008). Such studies have demonstrated the existence of obligate and facultative mutualists displaying functions ranging from nutrition, protection against biotic or abiotic stresses, to symbiont-manipulating reproduction regimes. The local biotic environment of these bacteria may promote speciation as a result of reproductive and ecological isolation (Moran *et al.* 2008). These studies thus (1) address new questions of co-evolution and macroevolution, and (2) further our understanding of the responses of the partnership to biotic or abiotic environmental stresses.

To date, functional and mechanistic objectives have not taken into account variation at the population level although this information is generally accessible in a number of environmental genomics projects. Usually, deep sequence coverage can detect single nucleotide polymorphisms (SNPs) and structural variations, such as copy number variants (CNVs) (Stranger *et al.* 2007), which can affect individual fitness. However, the field of population genomics (i.e. population studies analysing genome-wide genetic markers) is mainly developing apart from environmental genomics, despite the fact that the theoretical corpus of population genetics is well adapted to deal with environmental genomics data. Reciprocally, predictions and hypotheses can be derived from genomic neutrality tests of population differentiation due to environmental changes (i.e. population differentiation shown through association(s) between an environmental constraint and specific genetic markers). In this case, the genetic marker can be supposed to be a genetic trait of adaptation (Schmidt *et al.* 2008), which can thus be regarded and tested as a possible factor involved in individual fitness. This kind of idea may be considered as one of the purposes of comparative genomics or metagenomics projects.

**Figure 3:**
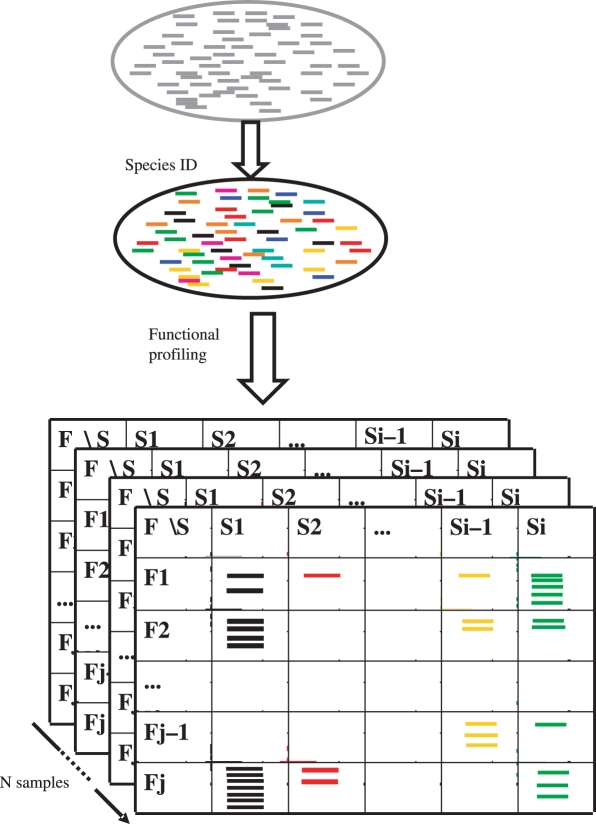
Spatio-temporal three-dimensional organisation of sequence-derived datasets. The set of environmental genomic, *cDNA*, or protein sequences (grey bars) is ascribed to a set of i Species (S), thus resulting in species-labelled sequences (colour bars). The aim of functional analysis and profiling is to ascribe species-labelled sequences to a set of j functional categories (F), thus resulting in a ‘potential function × species’ understanding of the ecosystem. The third dimension of the matrix corresponds to spatio-temporally replicated samples, such as samples subjected to various environmental constraints, or samples at different points in time. This kind of dataset can be analysed not only to understand the mechanisms induced by a forcing variable, but also to select and parameterize the components that have to be included in a model.

One major result of environmental genomics projects is the possibility of reconstructing and modelling potential metabolic and regulatory networks. However, these data cannot be readily used to formalise models of ecosystem functioning, as no data can be directly assigned to parameter variables: spatio-temporal variations must be taken into account if ecosystem functioning is to be comprehensively modelled from three-dimensional data matrices, as shown in Fig. 3. Experimental metagenomics, metatranscriptomics and metaproteomics projects testing the consequences of different environmental constraints on physico-chemical measurements can define the most important variables to include in a formal model of ecosystem functioning. Statistical modelling of a given ecosystem requires the kind of data presented in Fig. 3 and metadata, such as biogeochemical analyses, must be included to help the interpretations. It is also possible to model environmental genomics data from a stoichiometric approach or from a kinetic approach (e.g. Röling *et al.* 2007). Incorporation of spatio-temporal variations into the model would, in itself, lead to a change of scale. Even if environmental genomics is generally focussed at a small scale, it can be speculated that the data contain fractal properties of self-similarity (i.e. sub-units at multiple levels reflecting the structure of the whole object) and fractional dimensionality. These fractal properties could be tested to allow further rescaling at higher levels. As far as we know, such approaches have not yet been used. Such a model could in return be a source of testable hypotheses of ecosystem functioning, and could be used to predict changes in a given ecosystem.

## Glossary

cDNA: complementary DNA, reverse-transcribed or copied from an RNA template.
*ChIP-on-chip* approaches: a method combining Chromatine Immuno Precipitation (ChIP) with microarray (chip) technology to study interactions between proteins and DNA. Mainly used to determine the locations of binding sites, and to understand gene expression and regulation.
Environmental genomics: analysis of large-scale sequence-based information (such as DNA) obtained from a variety of environmental samples, at cell, organism, population, and community levels.
Environmental post-genomics: gene functional characterisation approaches and genome-wide expression analyses in an environmental context. Includes *transcriptomics* (analysis of the complete set of transcripts), mainly by mass sequencing of transcript-derived cDNA, through the development of second-generation sequencing machines, and *proteomics* (analysis of the complete set of proteins), mainly by coupled liquid or gas-chromatography/ tandem mass spectrometer (LC and GC-MS-MS) for *de novo* identification. Along with the metagenome, the *metatranscriptome* and the *metaproteome* can be analysed, when considering a community of organisms.
Fosmid: particular vector designed for the insertion of large-size DNA fragments.
Gene synteny: The order of genes on a chromosome region and its conservation.
Homologous and heterologous identification: similarity-based analysis of unknown genes by comparison with same-species (homologous) or cross-species (heterologous) sequences.
Insert: a DNA fragment of interest that has been cloned within a vector, such as a plasmid (non-chromosomal bacterial DNA) or a fosmid.
Large insert libraries: collections of cloned DNA inserts of long size (>20 000 bp).
Metagenomics: analysis of a mixed set of genomes from a community of organisms.
Microarray, DNA array, DNA chip: membrane or glass-slide surface where known DNA sequences are fixed, each at specific XY coordinates, to act as anchors for their complementary sequences. Mainly used for simultaneous expression surveys of a great number of genes.
Microfluidics: devices designed to manipulate and analyse microliter volumes of fluids in order to assay the composition within small samples.
Open reading frame: nucleotide sequence located between a start codon and a stop codon, thus potentially translatable into a polypeptide.
Orthologs: homologous genes found in different genomes and resulting from the duplication of an ancestral sequence during a speciation event.
Overexpression: molecular biology method resulting in the enhanced expression of a gene of interest in order to characterise its product or its impact on phenotype
Paralogs: homologous sequences resulting from an internal duplication event and belonging to the same species genome.
Promoter: sequence region upstream of a gene, to which RNA polymerase binds for gene transcription, and involved in gene expression regulation. Promoters can work in concert with other regulatory elements.
Regulatory cis-acting element: short DNA sequence in a promoter interacting with transcription factors to regulate expression of the downstream gene.
Shotgun sequencing: random sequencing of a high number of short anonymous fragments of genomes

## SUPPORTING INFORMATION

**Box S1** Strategies in environmental genomics.

**Box S2** Selection of bioinformatics tools for analysing environmental genomics data.

As a service to our authors and readers, this journal provides supporting information supplied by the authors. Such materials are peer-reviewed and may be re-organized for online delivery, but are not copy-edited or typeset. Technical support issues arising from supporting information (other than missing files) should be addressed to the authors.
