# Supplementary material for: Integration of molecular functions at the ecosystemic level: breakthroughs and future goals of environmental genomics and post-genomics
Source: Ecol Lett. 2010 Jun;13(6):776–91. doi: 10.1111/j.1461-0248.2010.01464.x (PMC2901524; doi:10.1111/j.1461-0248.2010.01464.x)

Box 1 : Strategies in environmental genomics

Environmental genomics allows targeting of communities as a whole in environmental samples (community genomics, metagenomics) or of single (micro)organisms isolated from the environment (single cell genomics, organism genomics).

(1) Isolation : Cells belonging to a certain lineage can be separated from their community using a variety of techniques, coupled or not with Fluorescent *In Situ* Hybridization (FISH), such as flow cytometry (Zehr *et al*. 2008) or *micro-fluidics* (Marcy *et al*. 2007).

(2) DNA extraction : Genome analysis begins with the extraction of DNA from community samples, isolated cell(s) or individual(s). A Whole-Genome Amplification (WGA) should be done when required (e.g. single cells) to get enough DNA template for sequencing. However, this step can induce important amplification bias.

(3), (4) Library preparation and sequencing : Depending on the aims of the studies, two different approaches can be employed. Large size fragments of DNA can be cloned into vectors such as bacterial artificial chromosomes and *fosmids. S*creening of *large-insert* libraries allow to select clones bearing functionally or phylogenetically relevant genes before sequencing (function- / organism-centered strategy) (Béjà *et al.* 2000; de la Torre *et al*. 2003). This approach is now less used mainly because of the large amount of handwork needed to prepare and to screen libraries. In the second approach, also named *shotgun sequencing*, genomic DNA is fragmented into numerous, partially overlapping, small size fragments which are then randomly sequenced (environment-centered and function- / organism-centered strategy). This approach is well adapted for second generation mass sequencers. These machines based on new sequencing technologies yield a larger quantity of sequence information at a lower price than dye-terminator sequencing. Among these, the 454 Life Sciences pyrosequencer has proved to be useful in numerous studies in the various fields of ecology (http://www.454.com/publications-and-resources/index.asp).

(5) Bioinformatics analysis : Assembly softwares are used if possible to assemble sequences into larger fragments of genomes, or even near-complete genomes. It is noteworthy that, in populations with a high genetic diversity, assembly will be made of sequences originating from different cells and will represent composite genomes (Tyson *et al*. 2004; Venter *et al*. 2004; Zehr *et al*. 2008). Ultimately, bioinformatics analyses predict genes and identify their potential functions to determine diversity and functional potentialities of naturally occurring organisms and communities.

(6) Community and ecosystem integration : The integration of diversity and functional profiling at the ecosystem level requires various approaches that are complementary : (meta)transcriptomics, (meta)proteomics, physiological and biochemical characterisation, experimental analysis, microscale and large-scale environmental data measurements, mathematical modelling.

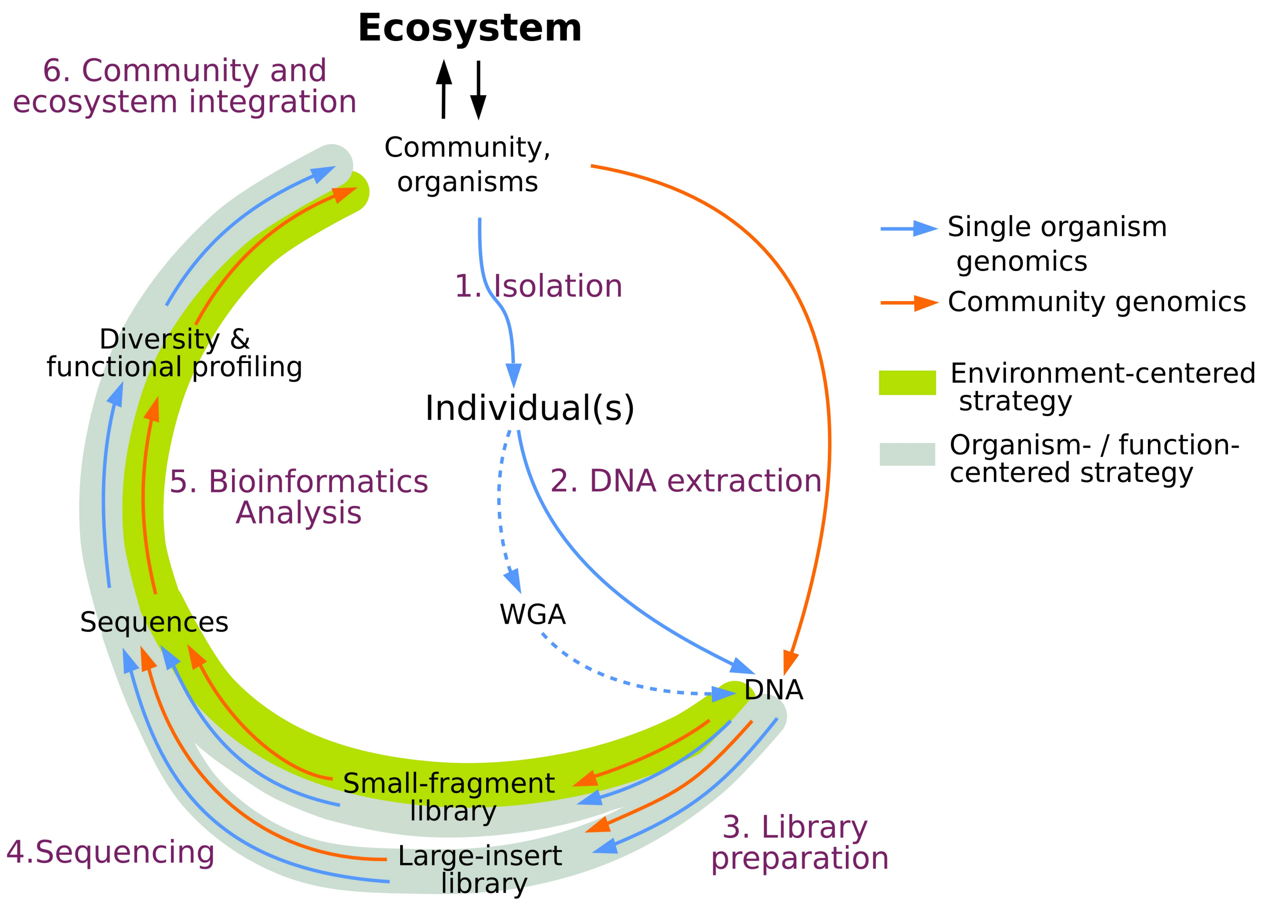

Supplement: Supplementary file 1 [file ele0013-0776-SD1.pdf]
