# Supplementary material for: Integration of molecular functions at the ecosystemic level: breakthroughs and future goals of environmental genomics and post-genomics
Source: Ecol Lett. 2010 Jun;13(6):776–91. doi: 10.1111/j.1461-0248.2010.01464.x (PMC2901524; doi:10.1111/j.1461-0248.2010.01464.x)
Supplement: Supplementary file 2 [file ele0013-0776-SD2.pdf]

Box 2 Selection of bioinformatics tools for analysing environmental genomics data

The advent of massive sequencing methods is yielding increasing amounts of genomic data and metadata. Sophisticated statistical analysis using dedicated databases is necessary to interpret these data. Various algorithms and websites have been developed in order to bring the power of these statistical analyses within the reach of scientists that are not bioinformatics specialists. Such analytical tools will enhance understanding of ecosystems if they are incorporated into robust ecological and statistical frameworks.

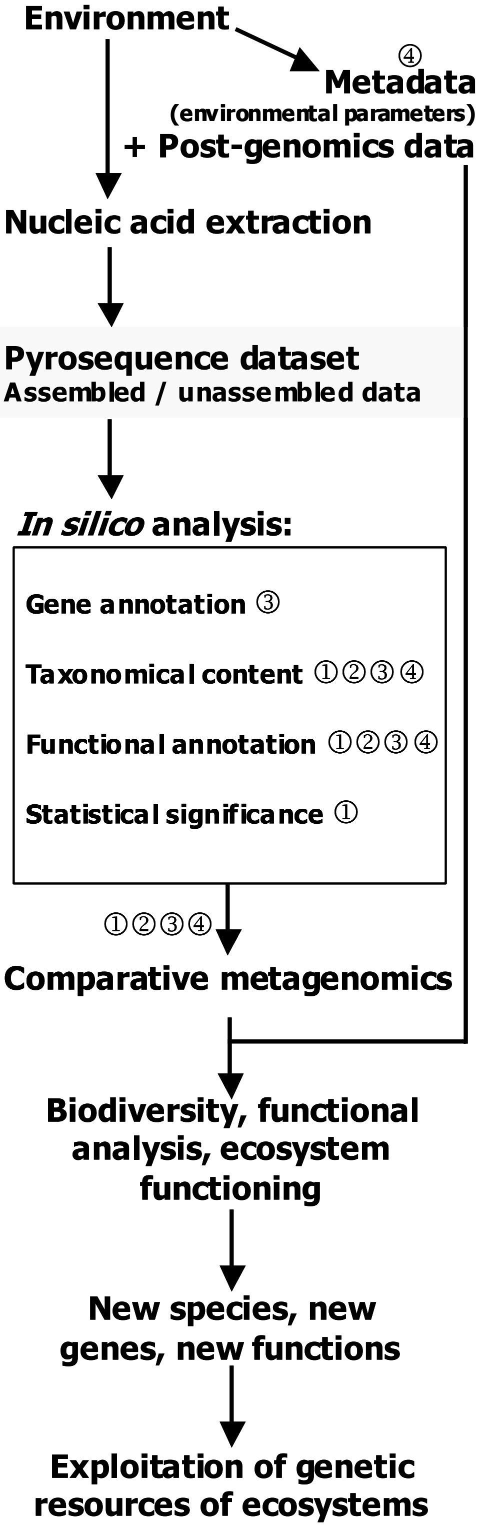

Many bioinformatics tools have recently been published. Most of these publications describe algorithms that enhance the robustness of statistical analysis at all levels of characterisation (species richness, functional richness, frequency of gene families, taxonomic classification, *de novo* genome assembly). MEGAN V3 (Metagenome Analysis Software) (Mitra et al. 2009), IMG/M (Integrated Microbial Genomes) (Markowitz et al. 2008), MG-RAST (Meta Genome Rapid Annotation using Subsystem Technology) based on the SEED framework (Meyer et al. 2008) and CAMERA (Community Cyberinfrastructure for Advanced Marine Microbial Ecology Research and Analysis) (Seshadri et al. 2007) are the main bioinformatics resources available online.

① MEGAN V3.6, a freely available software, estimates the taxonomical content by analysing BLAST (Altschul et al. 1997) comparisons of a set of reads against one or more reference databases, such as NCBI (National Center for Biotechnology Information) or genome-specific databases. The comparative analysis of the functional content of metagenome datasets is performed by tools using Clusters of Orthologous Groups of Proteins or Gene Ontology.

② IMG/M consists of 65 microbial community samples that are part of 20 metagenome studies and 5062 publicly available single microbial genomes data. The efficiency of genome analysis by IMG/M stems from this comparative context. This data management system provides tools for analysing the functional capability of metagenome data generated by shotgun sequencing.

③ MG-RAST is a fully-automated server dedicated to the annotation of metagenomes. It provides sequence annotation, phylogenetic classification and metabolic reconstructions. It accepts the upload of files directly in the format delivered by 454 pyrosequencing systems. The pipeline includes 394 completed public metagenomes from the curated SEED database and comparison tools.

④ CAMERA includes environmental metagenomic and genomic sequence data (43 datasets), associated environmental parameters (metadata), and precomputed search results, in order to carry out cross-analysis of environmental samples through BLAST search.

MEGAN V3: http://www-ab.informatik.uni-tuebingen.de/software/megan
IMG/M: http://img.jgi.doe.gov/cgi-bin/m/main.cgi
MG-RAST: http://metagenomics.nmpdr.org/
CAMERA: http://camera.calit2.net

REFERENCES

Markowitz, V.M., Szeto, E., Palaniappan, K., Grechkin, Y., Chu, K., Chen, I.A. *et al.* (2008). The integrated microbial genomes (IMG) system in 2007: data content and analysis tool extensions. *Nucleic Acids Res.*, 36, D528–D533.

Meyer, F., Paarmann, D., D'Souza, M., Olson, R., Glass, E., Kubal, M. *et al.* (2008). The metagenomics RAST server – a public resource for the automatic phylogenetic and functional analysis of metagenomes. *BMC Bioinformatics*, 9, 386.

Mitra, S., Klar, B. & Huson, D.H. (2009). Visual and statistical comparison of metagenomes. *Bioinformatics*, 25, 1849–1855.

Seshadri, R., Kravitz, S.A., Smarr, L., Gilna, P. & Frazier, M. (2007). CAMERA: A Community Resource for Metagenomics. *PLoS Biol.*, 5, e75.
